# NMR insights into the pre-amyloid ensemble and secretion targeting of the curli subunit CsgA

**DOI:** 10.1038/s41598-020-64135-9

**Published:** 2020-05-12

**Authors:** Lee Sewell, Fisentzos Stylianou, Yingqi Xu, Jonathan Taylor, Lea Sefer, Steve Matthews

**Affiliations:** Department of Life Sciences, Imperial College London, London, SW7 2AZ UK

**Keywords:** Biochemistry, Microbiology, Structural biology

## Abstract

The biofilms of *Enterobacteriaceae* are fortified by assembly of curli amyloid fibres on the cell surface. Curli not only provides structural reinforcement, but also facilitates surface adhesion. To prevent toxic intracellular accumulation of amyloid precipitate, secretion of the major curli subunit, CsgA, is tightly regulated. In this work, we have employed solution state NMR spectroscopy to characterise the structural ensemble of the pre-fibrillar state of CsgA within the bacterial periplasm, and upon recruitment to the curli pore, CsgG, and the secretion chaperone, CsgE. We show that the N-terminal targeting sequence (N) of CsgA binds specifically to CsgG and that its subsequent sequestration induces a marked transition in the conformational ensemble, which is coupled to a preference for CsgE binding. These observations lead us to suggest a sequential model for binding and structural rearrangement of CsgA at the periplasmic face of the secretion machinery.

## Introduction

The amyloid fibre is a unique biopolymer, in that it is comprised of protein subunits, and is stabilized through non-covalent interactions. Each subunit characteristically forms a stacked *β*-sheet that coalesces perpendicular to the fibril axis. Almost all residues in the core of the fibre contribute to its stabilisation, giving it a tensile strength similar to steel and other inorganic materials^1^. These remarkable material properties make the turnover of amyloid in biological systems a challenge, with its accumulation a causative factor in a number of pathologies, particularly neurodegeneration^2^. Moreover, studies have also shown that early oligomeric intermediates formed during amyloidogenesis are often particularly cytotoxic^3^. Despite the inherent risk of cytoxicity, many organisms have evolved mechanisms to utilise the amyloid fold. These ‘*functional amyloids*’ exist in all domains of life, and operate in a host of diverse roles^4^. Several bacteria utilise these fibres as a major component of their biofilm matrix, and have evolved distinct apparatus to co-ordinate secretion^5–7^, The first identified of such biofilm functional amyloids, and the best studied, is that of *Escherichia coli*^8,9^, The amyloid produced by this system, termed curli fibres, has been identified in a wide range of *Enterobacteriaceae*, with homologous systems identified in many Gram negative phyla^10,11^, Whilst providing bacteria with a structurally re-enforced scaffold, curli has also been implicated in pathogenic infection^12^, triggering host auto-immune responses^13^, and even accelerating neurodegeneration^14^. As a consequence of its extensive study, curli is a model functional amyloid system, driving research forward in a diverse range of applications, including the generation of small molecule inhibitors^15^ and implementation in novel biomaterials^16^.

**Figure 1:**
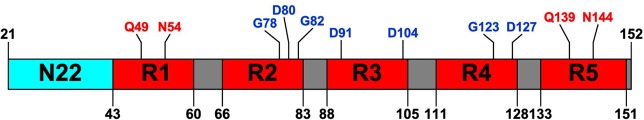
Schematic representation of the mature CsgA polypeptide. The amyloid core repeat regions R1-R5 (red) are flanked by linker regions (grey). Residues annotated in red are located in the most amyloidogenic repeats (R1 and R5), and have been reported to be critical for curli formation^40^. Residues annotated in blue act as gatekeeper residues that act to reduce the rate of CsgA aggregation prior to templating at the cell surface^41^. Numbering includes the signal sequence that is cleaved after perisplamic translocation.

Curli is assembled and regulated by the seven curli-specific genes (*csg*), found on two divergently transcribed operons, *csgBAC* and *csgDEFG*. The master regulator of biofilm formation, transcription factor CsgD, provides tight regulation of amyloid production in response to environmental conditions^17,18^, CsgA is the main component of the curli fibre, composed of 5 repeat regions (R1-R5) that are predicted to form the amyloid fold^19^ (Fig. 1). These repeats are 19–23 residues long and possess the consensus sequence Ser-X_5_-Gln-X_4_-Asn-X_5_-Gln. CsgA is exported into the periplasm through the SecYEG complex, as a 13.1 kDa mature peptide, and is maintained as an intrinsically disordered protein (IDP) by the chaperone protein CsgC^20^. It also possesses an N-terminal sequence (N_22_) that targets it for secretion across the outermembrane^21^. This pre-fibril state of CsgA is shuttled to the secretion complex, where it binds to CsgG, the outermembrane pore. CsgG forms a nonameric, 36-stranded *β*-barrel structure, with a > 1 nm pore that is gated at the periplasmic face^22–24^, CsgE and CsgF help co-ordinate CsgA translocation at either side of the membrane. CsgE localizes at the periplasmic face of the CsgG secretion pore, also forming a nonameric ring, which functions to prevent non-specific subtrate secretion^25,26^, and chaperones CsgA through the CsgG pore^22^. CsgF forms a tight complex with the extracellular face of CsgG, where it co-ordinates templating of curli fibres via an interaction with the nucleator CsgB^27–29^,

The amyloid fold of CsgA within the curli fibre has been extensively characterized, using data from a wide range of biophysical techniques^30–32^, Complementing experimental work on the fibrous state of CsgA, a model of CsgA nucleation and assembly has been proposed using ThT-binding studies^33^, and H/D exchange experiments have identified the most important repeats for driving amyloid formation^34^. Whilst these have provided valuable insights into fibre assembly, structural analysis of the pre-amyloid state of CsgA present within the perisplam has been more difficult. This is largely due to its disordered and transient nature. Indeed, much of our understanding of CsgA as an IDP comes from investigating other *csg*-encoded proteins and their relationship with CsgA. Previous work revealed a transient electrostatic mechanism for CsgC inhibition of CsgA aggregation^35^. Similarly, studies from the CsgE perspective have also highlighted the importance of transient electrostatic interfaces in controlling CsgA polymerization^36^. Studies have also shown that CsgA can cross seed homologs from other bacterial species, which raises the notion that curli fibers may facilitate multispecies biofilms^37^. The seeding specificity of curli is a more general phenomenon, as CsgA is able to alter the fibrillation kinetics of several human amyloidogenic proteins^38^.

This study provides new structural insights into the pre-amyloid CsgA ensemble and its targeting to the curli secretion system using solution-state nuclear magnetic resonance (NMR) spectroscopy. Structural features adopted by CsgA in solution were characterised by gleaning information from chemical shifts and transverse relaxation. These highlight the importance of prion-like motifs as points of conformational exchange in the repeat regions, and the abundance of polyproline II (PPII) in the disordered ensemble. Removal of the N-terminal targeting region (N_22_) causes a significant conformational redistribution within the repeat regions of CsgA, particularly near important regions that gate-keep amyloid formation, suggesting a regulatory role. Furthermore, we show that CsgE recognises the truncate in preference to the mature protein that includes N_22_. While the extrapolation of our conclusions to the bacterial cell has limitations, as we ignore the influence of other cellular factors, our work supports a model in which a step-wise progression of structural re-arrangements in CsgA occur during export and secretion. First, intramolecular interactions involving N_22_ together with CsgC assist in preventing inappropriate amyloid formation and premature binding of CsgE. Then, N_22_ is bound specifically by the periplasmic face of outermembrane CsgG where it is sequestered from pre-fibrillar CsgA. Finally, this alters the conformation distribution of the CsgA ensemble and primes CsgA for interaction with CsgE. Subsequent interaction with of the CsgE ring with the base of the CsgG pore drives encapsulation of CsgA and entropic release through the pore.

## Results

### Backbone assignment of the disordered, pre-fibril CsgA

Prior characterisation of CsgA by solution-state NMR had been hampered by low signal intensity, spectral overlap, and limited life-time of samples. To address these issues, we optimised several aspects of sample and experimental conditions. Low sensitivity is largely a consequence of low sample concentrations, typically sub 20 μM. We chose to use C-terminally His-tagged CsgA for rapid purification and increased yields. Previous studies have shown that his-tagged and untagged CsgA behave similar in amyloid aggrateion assays^39^.As efforts to increase the sample concentration by centrifugal methods promotes amyloid formation and precipitation, induction times for expression were lengthened in order to produce protein at higher intracellular concentrations. To abate fibril formation and also mimic the periplasmic state of CsgA, the curli inhibitory chaperone CsgC was added to samples at a 1:40 ratio. ^1^H-^15^N HSQC spectra were recorded in the presence and absence of CsgC, revealing no prominent chemical shift perturbations, consistent with previous work revealing the transient nature of the CsgC-CsgA encounter^35^ (Supplementary Figure S1). Further improvements were obtained by the reduction of sample temperature to 10 °C, which also favourably reduced the rate of unwanted amyloidogenesis. Use of higher strength magnetic fields (950 MHz) improved resolution for regions with the limited dispersion that is typical of IDPs. Final samples were 150 μM and stable for several days.

**Figure 2:**
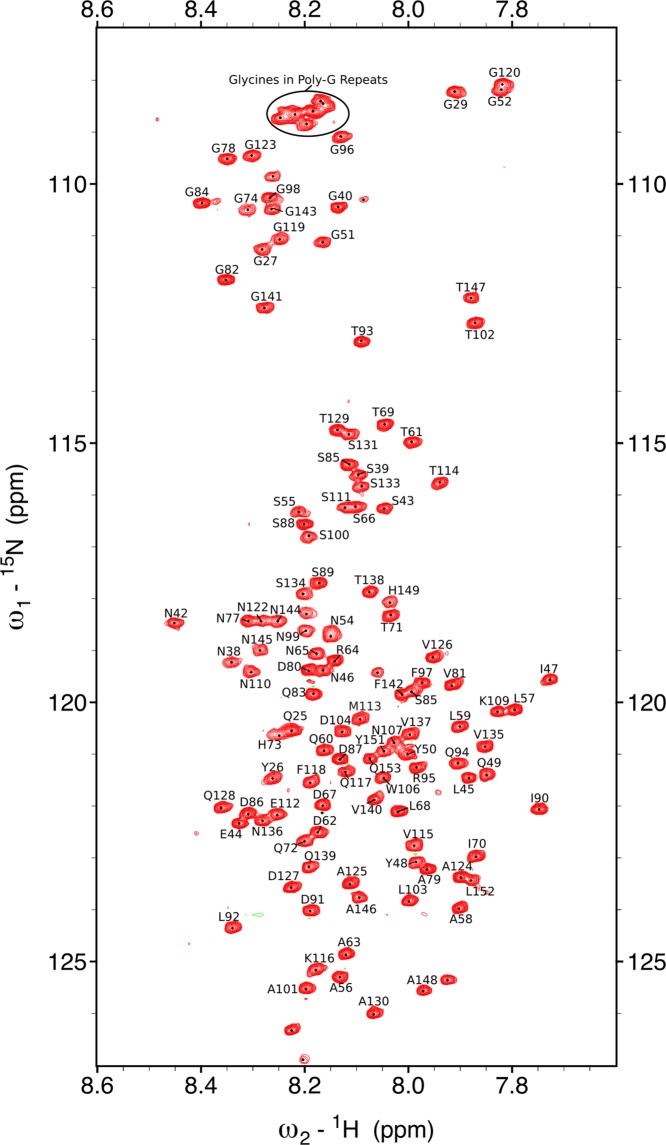
^15^N-^1^ H HSQC spectrum of 150 μM CsgA in 20 mM Na_2_ HPO_4_, pH 7.2, 10 °C. Amide resonances are labelled with one-letter code. Spectra recorded at 950 MHz. Overlapped glycine residues in glycine-rich repeats are highlighted at the top of the spectra. Chemical shifts are displayed in parts-per-million (ppm) units.

Assignment experiments were carried out using triple resonance methodology, with the hNcocaNNH pulse sequence particularly useful for overcoming ambiguity in overlapped regions. Despite narrow ^1^H chemical shift dispersion (7.6–8.4 ppm), ^1^H-^15^ N correlations were assigned for 118/134 (88.1%) of residues (Fig. 2). The C-terminal His-tag is excluded from this count. A majority of the unassigned residues are located in, or proximal to, the glycine-rich repeats within the N_22_. Prolines, P24 and P41, are also unassigned, as are residues located after the N-terminal methionine (G21-V23).

### The N_22_ region influences the conformational ensemble of CsgA

**Figure 3:**
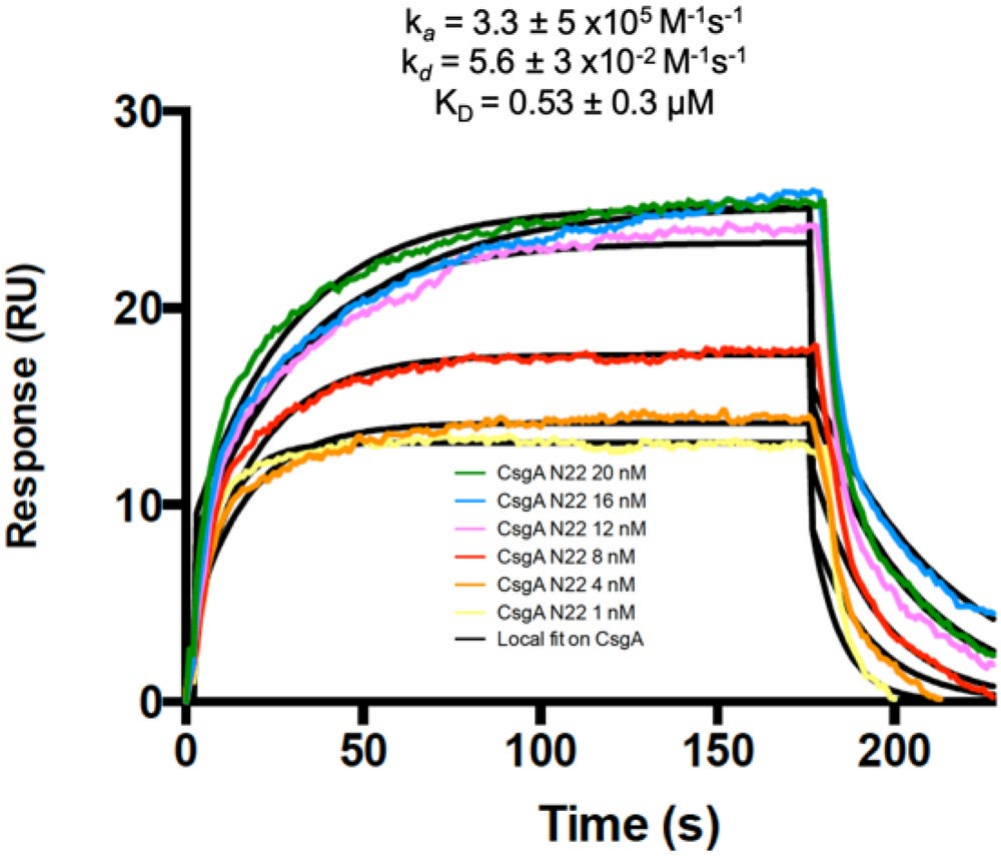
SPR sensorgrams obtained for CsgA N_22_ peptide indicate binding to CsgG. Binding of the CsgA N22 peptide was assessed in the range of 20 − 1 nM concentrations using a non-activated flow cell as a control. The binding curves were fitted based on 1:1 binding model and the parameters calculated locally. Final value of the affinity constant obtained was 0.53 μM.

Next, using our backbone assignment, we sought to characterize the curli pathway from the perspective of CsgA. The binding affinities of the export complex were assessed using surface plasmon resonance (SPR). In this experiment, an NTA sensor chip was activated using Ni^+^ and the CsgG-His complex was immobilized via the His-tag, with a peptide comprising N_22_ from CsgA being injected. Experiments with full length CsgA were not successful, due to the aggregation of CsgA on the SPR chip. Our N_22_ peptide data showed a specific interaction with a dissociation constant in the micromolar range (0.53 μM; Fig. 3). This is broadly consistent with a value of 28.3 μM, recently measured for the shorter N_6_ peptide using isothermal calorimetry. The CsgG-N_22_ interaction likely reflects the specific interaction with full-length CsgA, as it has been shown the N_22_ peptide is not part of the amyloid fold and it is sufficient to direct secretion of heterologous proteins to the cell surface^21^. The interaction between the N_22_ and CsgG would therefore sequester the N-terminal region from the dynamic solution state ensemble. Therefore, to probe differences in the conformational preference of CsgA between sequestered and un-sequestered N_22_, we produced a truncation of CsgA lacking this region (CsgA_ΔN22_).

**Figure 4:**
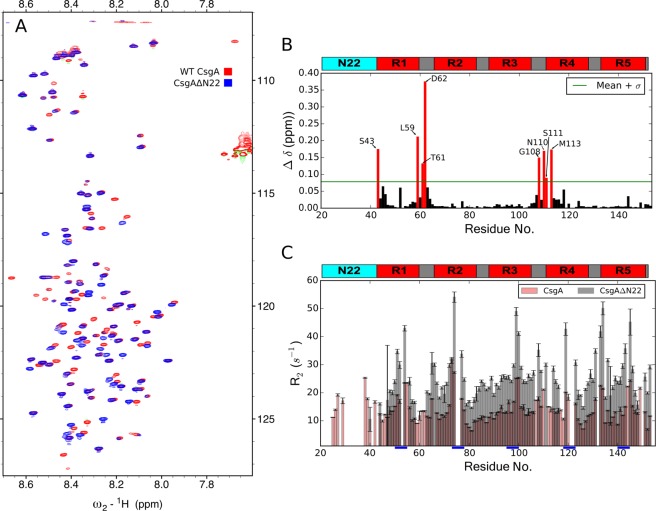
Truncation of the N_22_ region shifts dynamics in key amyloidogenic regions. **(A)** Overlay of ^15^N-^1^ H HSQC spectra of CsgA (red) and CsgA_Δ*N*22_ (blue) highlights widespread CSPs. **(B)** Mapping of these CSPs to the sequence reveals two prominent clusters that are proximal to key gate-keeper residues. The threshold (green line) for selection of most significant shifts (Δ δ) was calculated as the mean of all CSPs plus the standard deviation. **(C)** Transverse relaxation rates are elevated across the sequence of the truncation. The highest relaxation rates in both truncate and full-length are noted proximal to prion-like repeats (underlined blue). Error bars represent the estimated errors from fitting exponential decay from experiments performed in triplicate. Bar plots are transparently overlapped according to colouring in the legend to illustrate elevated relaxation in CsgA_Δ*N*22_.

The ^1^H-^15^ N HSQC spectra of CsgA_ΔN22_ reveals significant chemical shift differences (Fig. 4A), which indicates changes in the conformational distribution of the disordered ensemble. In order to map these changes to the CsgA sequence, independent assignment experiments were carried out for CsgA_ΔN22_ and chemical shift perturbations (CSPs) plotted against the CsgA sequence (Fig. 4B). Outside of shifts proximal to the N_22_ region, which would be a direct result of the truncation, two main clusters are observed where the largest changes occur. Residues L59, T61, and D62 are located within repeat R1, which is essential for amyloid formation^40^. Interestingly, G108, N110, S111, and M113 are located in the linker between the amyloidogenic R3 and the non-amyloidogenic R4^40,19^, and are flanked by gatekeeper residues D91, D104, G123 and D127, which prevent the repeat regions from participating in nucleation events^41^.

To further investigate differences between the molecular ensembles of CsgA and CsgA_Δ*N*22_, transverse relaxation rates (R_2_) were measured for the amide resonances. This parameter is sensitive to conformational dynamics on the fast time scale (ps-ns), and also influenced by slower exchange processes (μs-ms)^42^. It is often used to identify areas of backbone flexibility and conformational exchange processes. The R_2_ rates of CsgA are generally low across the sequence, in the range of 10 s^−1^, typical of an IDP due to the inherent and high flexibility (Fig. 4C). In contrast, CsgA_ΔN22_ has significantly elevated R_2_ values across the whole sequence, approximately twice that for CsgA, suggesting that the truncate exhibits conformational exchange on a μs-ms time scale. In order to probe for μs-ms dynamics further, CPMG experiments were conducted on both samples, however no dispersion was detected. The dynamics indicated by the increased R_2_ values suggest they may arise from intramolecular conformational exchange or self association on the faster end of the μs timescale. A few short sequence regions of CsgA, proximal to the glycine-rich motifs of repeats, exhibit elevated transverse relaxation (as denoted by the blue bars in Fig. 4C). A previous study identified these hexapeptide motifs (Q-X-G-G/F-G-N) as similar to to the highly amyloidogenic peptide regions of animal and yeast prion proteins, and demonstrated they are vital for curli formation^43^. The R_2_ values observed here appear to support their findings, and highlight that important structural transitions appear to occur in these motifs. Although it has been shown *in vitro* that both CsgA_Δ*N*22_ and CsgA readily formed amyloid fibrils on broadly similar timescales, the aggregation kinetics display different protein concentration dependencies^44^.

**Figure 5:**
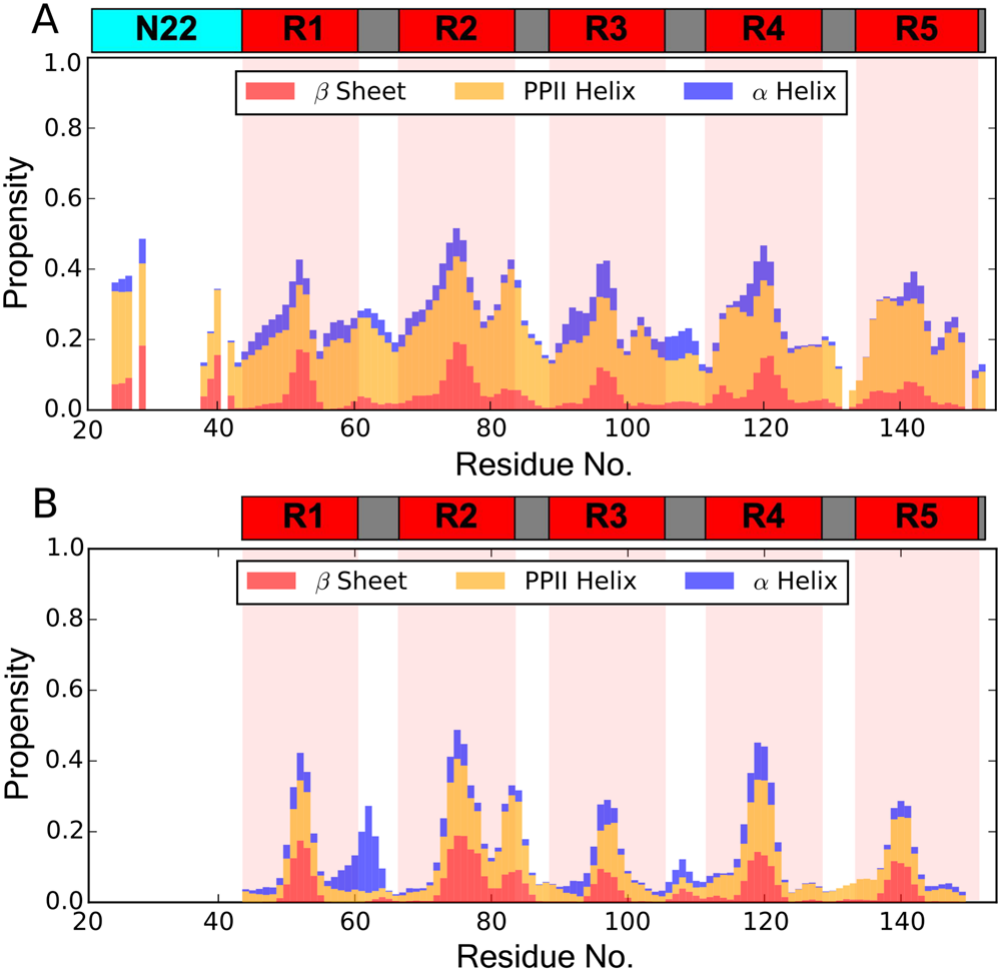
Residual secondary structure propensities (SSPs) in CsgA transition away from PPII helices upon truncation of N_22_. **(A)** Outside of random coil, full-length CsgA displays a predominantly PPII helix structure. Small *β*-sheet propensities are noted in the repeat regions. **(B)** CsgA_Δ*N*22_ has a reduced PPII helix density, with a significant α-helix propensity appearing between R1 and R2. SSPs are represented by stacked bar plots, with colours showing the relative propensity at each residue, as in the legend. Random coil propensity, the dominant structure in IDPs, is not actively displayed. Regions highlighted red on the plots align with the repeat regions in the sequence schematic above.

To better gauge how differences in the chemical shifts and R_2_ relaxation rates related to the conformational distribution in the CsgA/CsgA_Δ*N*22_ ensembles, secondary structure propensities (SSPs) were calculated. We used the δ 2D method to calculate propensities from chemical shift data, as it is optimally parameterized for disordered proteins. As would be expected for IDPs, the major SSP in CsgA is random coil, however there are noticeable sub-populations of other motifs (Fig. 5A). In full-length CsgA, the major non-coil propensities are PPII helices. This type of secondary structure has high conformational flexibility, providing a low energy barrier for conversion to other structural motifs, and has been observed as an important intermediate in transitions from disordered states into the amyloid fold^45,46^, Truncation of the N_22_ causes a transition away from PPII, particularly in the linker regions between the repeats (Fig. 5B). Furthermore and most notably, a helix propensity emerges between R1 and R2, providing a structural basis for these the prominent CSP clusters.

### CsgE interactions with CsgA

We proceeded to monitor the interaction between both CsgA constructs and CsgE, the periplasmic cap to the CsgG pore, from the perspective of CsgA. Previous studies have a identified weak and transient interactions between CsgA and CsgE when studying CSPs from the CsgE perspective^36^. Our rationale was that, because the N_22_ region binds to CsgG, and we have identified conformational re-arrangement in CsgA_Δ*N*22_, mimicking sequestration, CsgE may differentially bind to the two CsgA species. We adopted the same approach by employing the oligomerization-disrupted W48A/F79A CsgE mutant as it gives markedly better NMR spectra, while retaining is functional chaperoning effect on CsgA^26^. The W48A/F79A CsgE mutant was incubated at a four-fold excess to CsgA prior to recording spectra. Small but significant chemical shift changes were noted for CsgA_Δ*N*22_ (~0.015 ppm) (Supplementary Figure S2).

**Figure 6:**
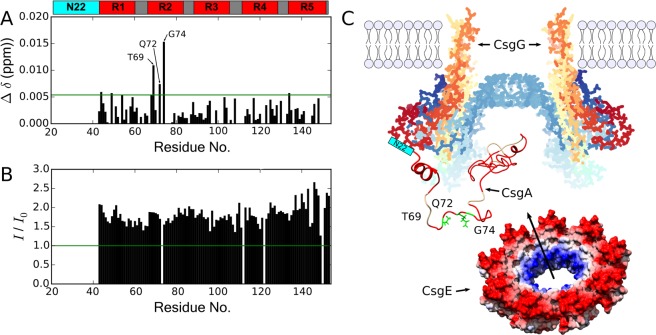
CsgE interactions are only observed with CsgA_Δ*N*22_ truncate, which stabilises the pre-fibril state prior to secretion through into the extracellular space. **(A)** CsgA_Δ*N*22_ CSPs when incubated with a four times excess of W48A/F79A CsgE. Despite observed differences being relatively small, no significant shift differences were noted when CsgE was incubated with full-length CsgA. This suggests a transient interaction in the region adjacent to the α-helix propensity that emerged in the truncate. **(B)** Differences in signal intensity between CsgA_Δ*N*22_, incubated overnight at 4 °C, with a four times excess of CsgE (*I*), and without (*I*_0_). Signal loss likely a result of transition to an amyloid state. CsgE reduces the rate of this signal loss. **(C)** Model of key interactions during the periplasmic export of CsgA. The N_22_ region binds to CsgG, causing a structural re-arrangement of CsgA. CsgE acts as a nonameric cap, preventing amyloid formation and facilitating export through transient interactions with CsgA in the region of T50/Q53/G55. This then results in CsgA being capped in the pore by the CsgE ring, with release of N_22_ and secretion through CsgG. Sequence motifs of CsgA coloured as in the sequence schematic in (**A**), CsgE surface coloured according to electrostatic potential - positive (red) through to negative (blue). PDB accession codes = CsgE: 2NA4, CsgG: 4UV3.

Strikingly, the significant shifts (T69/Q72/G74) are located at the start of R2 (Fig. 6A). As this cluster is proximal to where the α-helix propensity appears in CsgA_Δ*N*22_, it suggests this conformational transition upon CsgG binding to N_22_ may be important for CsgE recognition. Further supporting this, no significant shifts of this magnitude were observed in the incubation of CsgE with full-length CsgA (Supplementary Figure S3).

Previous work has shown the CsgE has a stabilising effect of the disordered CsgA monomer and this effect is driven by charge-charge interactions^36^, akin to the effect of CsgC on amyloidogenic proteins^35^. Notably, charge swap mutations of the E31 and E85 in CsgE increased CsgE-mediated stabilization of CsgA. We also observed the stabilising effect of CsgE on the unfolded state of CsgA_Δ*N*22_. After 24 hours incubation at 4 °C, samples with CsgE present produced greater signal intensity (Fig. 6B). As the amyloid fibre is not visible in solution state NMR, higher signal intensity relates to a higher population of soluble pre-amyloid CsgA species. This is consistent with the hypothesis that CsgE has a similar function to CsgC in preventing premature fibre formation in the periplasm.

## Discussion

The rapid polymerization of CsgA into the functional curli amyloid is critical for construction of the *E. coli* biofilm. However, whilst the time-scale for this process favours the organism, it presents a challenge for structural studies. In this work, we addressed this challenge by optimising solution conditions and present an NMR characterisation of the CsgA pre-amyloid state, both prior to and after capture by the secretion machinery. The utilisation of the NMR assignment has allowed us to observe structural motifs within the intrinsically disordered monomer. Non-coil secondary structure propensities in free CsgA favour the PPII conformation that has been found important in amyloid transitions^45,46^, As expected, there are minimal *β*-sheet propensities that are concentrated in the core-forming repeat regions. Transverse relaxation rates are elevated in the prion-like motifs of the repeat regions, suggesting these are important sites of conformational exchange en route to amyloid precipitation. Our work has shown that the N-terminal N_22_ region binds specifically to CsgG, and the structural details of this has been recently illuminated with the cryo-electron microscopy structure of the CsgG-CsgF-CsgAN_22_ complex^47^. A six residue stretch (V22-G27) is bound in a surface channel on the periplasmic face of CsgG, with conserved residues V23, Q25, and Y26 making the most important contacts. We sought to characterize the conformation upon recruitment to the secretion pore, and therefore produced a truncate - CsgA_Δ*N*22_. Widespread chemical shift differences in CsgA_Δ*N*22_ suggested a switch in the disordered ensemble, concentrated in clusters of residues previously identified as gatekeepers for prevention of amyloidosis. Further characterisation of the truncate revealed transverse relaxation was approximately twice the rate compared with the full-length protein, and also that PPII propensity collapses, coupled with the emergence of a prominent α-helical motif between R1 and R2. These conformational perturbations suggest that the N_22_ has a dual-purpose, first directly targeting CsgA to CsgG and once bound by CsgG inducing a conformation transition in CsgA that facilitates secretion through the CsgG pore.

It is important to note that the N_22_ region is not cleaved from CsgA, therefore a mechanism must exist for its release from the periplasmic face of CsgG prior to secretion. Indeed, the structural transitions observed in our truncate provide new insight into how this may occur. Interactions with CsgE, which regulates periplasmic substrate export, were observed from CsgA_Δ*N*22_, but was not detectable with full length CsgA. Therefore, combining results from this study with the structure-function analysis of CsgE^36^, we present a model for CsgA recognition and the interplay with the CsgE cap (Fig. 6C). In short, we propose that CsgA is kept in it monomeric state with in the perisplasm by freely diffusing CsgC, until it is recruited to CsgG via the specific interaction with its N-terminal N_22_ region. Subsequent sequestration of N_22_ induces a conformational re-distribution of the disordered CsgA ensemble, which promotes interactions with CsgE and could also assist in stabilising the CsgE nonamer. This notion is also consistent with recent pull-down assays showing an interaction between CsgE and CsgA_Δ*N*22_, but not CsgA^47^. The CsgE oligomer then plugs the periplasmic aperture of the CsgG pore and releases the N_22_ region. Transient electrostatic interactions from CsgE stabilise the unfolded state of CsgA in its captured state preventing premature amyloid accumulation. Furthermore, oligomerised CsgE encloses the CsgA substrate within the CsgG-CsgE vestibule, reducing its conformational flexibility and the entropy. The release of this high energy intermediate to the extracellular milieu contributes to the driving force for secretion.

A persistent feature of pre-amyloid in curli and other functional amyloid systems is the role of weak transient interactions in preventing amyloid formation and guiding the protein for export and subsequent templating^35,36,48–50^, The transience of interactions between CsgC/CsgE and CsgA, as observed by fast exchange behaviour in NMR titration experiments^35,36,49^, is likely important for the progressive handling and delicate manipulation of the dynamic pre-amyloid ensemble. Such fine tuning of this early stage of amyloid formation enables the bacteria to deliver subunits in the appropriate state for efficient and homogeneous fibre format at the surface. It also explains why knock-out mutants of AgfE and AgfC in *Salmonella typhi* do not abrogate secretion but assemble mixed fibre morphology^51^.

## Methods

### Protein expression and purification

The gene encoding the mature CsgA polypeptide (Uniprot P28307, residues 22–151) was obtained by PCR amplification of *E. coli* BL21 (DE3) genomic DNA. An expression construct with a C-terminal His-tag was obtained by ligation into a pET-28a vector using *NcoI*/*XhoI* sites. The CsgA_Δ*N*22_ truncation was cloned using the Q5 Site-Directed Mutagenesis kit (NEB). CsgA variants were transformed into BL21 (DE3) *E. coli* expressions strains, inoculated into 800 mL of labelled M9 minimal media (^15^NH_4_Cl/^13^ C_6_ D-Glucose), and grown to 1.0 OD_600_. Temperature was decreased to 22 °C, IPTG (isopropyl-BD-thiogalactoside) added to a final concentration of 1 mM, and expression induced for 16 hours. Harvested cell pellets were stored at −80 °C. The pTrc99A expression vector for oligomerization-inhibited W48A/F79A CsgE mutant was kindly provided by Professor Matthew Chapman. W48A/F79A CsgE was purified as outlined previously^52^. CsgC-6xHis and CsgG-6xHis were expressed and purified as described previously^53^.

### Denaturing purification of recombinant pre-amyloid CsgA

Cell pellets were resuspended in 15 mL g^−1^ of denaturation buffer (50 mM Tris- HCl, 6 M guanidine hydrochloride, pH 8). Lysis was achieved using sonication, and cell debris removed by centrifugation at 30,000 RCF for 30 minutes. Supernatant was loaded onto a 5 mL FF HisTrap column (GE Life Sciences) that was pre-equilibrated in denaturation buffer. Immobilized metal affinity chromatography (IMAC) was conducted in a step-wise manner with increasing imidazole concentrations (30/60/120/300 mM imidazole, 50 mM Tris-HCl, 6 M guanidine hydrochloride, pH 8). Fractions containing pure amyloid monomer were determined using SDS-PAGE, then pooled, concentrated to 2.5 mL using 10 kDa cut-off Vivaspin 20 device (Sartorius Biotech), and buffer-exchanged by passing through a PD-10 desalting column (GE Life Sciences) equilibrated in low salt buffer (20 mM Na_2_PO_4_, pH 7.2). Samples were either immediately used, or flash frozen in liquid N_2_ and lyophilised.

### NMR data collection, assignments, and analysis

Samples of 150 μM CsgA, [U-^15^N] or [U-^13^C, ^15^N], were prepared in low salt buffer (20 mM Na_2_ PO_4_, 10% D_2_ O, pH 7.2. To prevent CsgA from transitioning to amyloid in the time course of assignment experiments (several days), curli-inhibitory chaperone was added at a concentration of 3.75 μM.

All spectra were recorded at 283.2 K. Data were collected on either a Bruker Avance III HD 950 MHz spectrometer, or a Bruker Avance III HD 800 MHz spectrometer. Both were equipped with a 5 mm TCI cryoprobe. Assignment of C α, C β, H^N^, and N chemical shifts were achieved using the following triple resonance spectra: HNCACB^54^, HNcoCACB^54^, hNcocaNNH^55^. Non-uniform sampling (NUS) methodologies were implemented in data collection. All spectra were processed with NMRPipe^56^, using SMILE to process NUS data^57^. Spectra were visualised and analysed using SPARKY^58^. Semi-automated, iterative backbone assignment was achieved using the algorithm MARS^59^. Secondary structure populations were determined from assigned chemical shifts, using the δ 2D methodology that is optimized for analysing disordered proteins^60^. ^15^N transverse relaxation parameters were acquired using Carr-Purcell-Meiboom-Gill (CPMG) experiments^61^. These experiments were recorded with 14 different CPMG frequencies, ranging from 50 to 1600 Hz with three points repeated in duplicate, with a 20 ms mixing time per CPMG block. Values of R_2_ were derived from line shape integration of peaks and fitting using the software package NMRPINT^62^. For interaction experiments, CsgA constructs (100 μM) were incubated with W48A/F79A CsgE (400 μM) for 24 hours at 4 °C prior to recording spectra. No CsgC was included in these samples. Receiver gains were set to the same value. CsgE was buffer exchanged into low salt buffer immediately before incubation. Chemical shift perturbations were calculated as a euclidean distance (*d*), and weighted as previously published^63^.

### Surface plasmon resonance

The experiments were performed on a Biacore 3000 using an NTA sensor chip (GE Healthcare, USA). The running buffer (10 mM HEPES, 150 mM NaCl, 0.36 mM DDM, pH 8.0) and the remaining buffers were prepared according to manufacturerâ€™s instructions, using 0.36 mM DDM as surfacant substitute. The senor chip was activated with 30 μL of 0.5 mM NiSO_4_ at 10 μl/min. 30 μL of 200 nM CsgG were immobilized at 10 μl/min. 150 μl and 250 μl of N_22_ peptide was injected at 50 μl/min at varying concentration. The reference flow cell was a non-activated surface, as recommended by GE Healthcare. Curve fitting was done based on a 1:1 binding model.

## Supplementary information

Supplementary Information.

## Data Availability

Accession codes CsgA chemical shift assignment have been deposited under Biological Magnetic Resonance Bank [BMRB] ID codes 50138.
