# Supplementary material for: NMR insights into the pre-amyloid ensemble and secretion targeting of the curli subunit CsgA
Source: Sci Rep. 2020 May 12;10:7896. doi: 10.1038/s41598-020-64135-9 (PMC7217966; doi:10.1038/s41598-020-64135-9)
Supplement: Supplementary file 1 — Supplementary Information. [file 41598_2020_64135_MOESM1_ESM.pdf]

Supplementary Information

NMR insights into the pre-amyloid ensemble and secretion targeting of the curli amyloid subunit CsgA

Lee Sewell[1], Fisentzos Stylianou[1], Yingqi Xu[1], Jon Taylor[1], Lea Sefer[1], and Steve Matthews[1]*,

[1]Department of Life Sciences, Imperial College London, London, SW7 2AZ, UK
*s.j.matthews@imperial.ac.uk

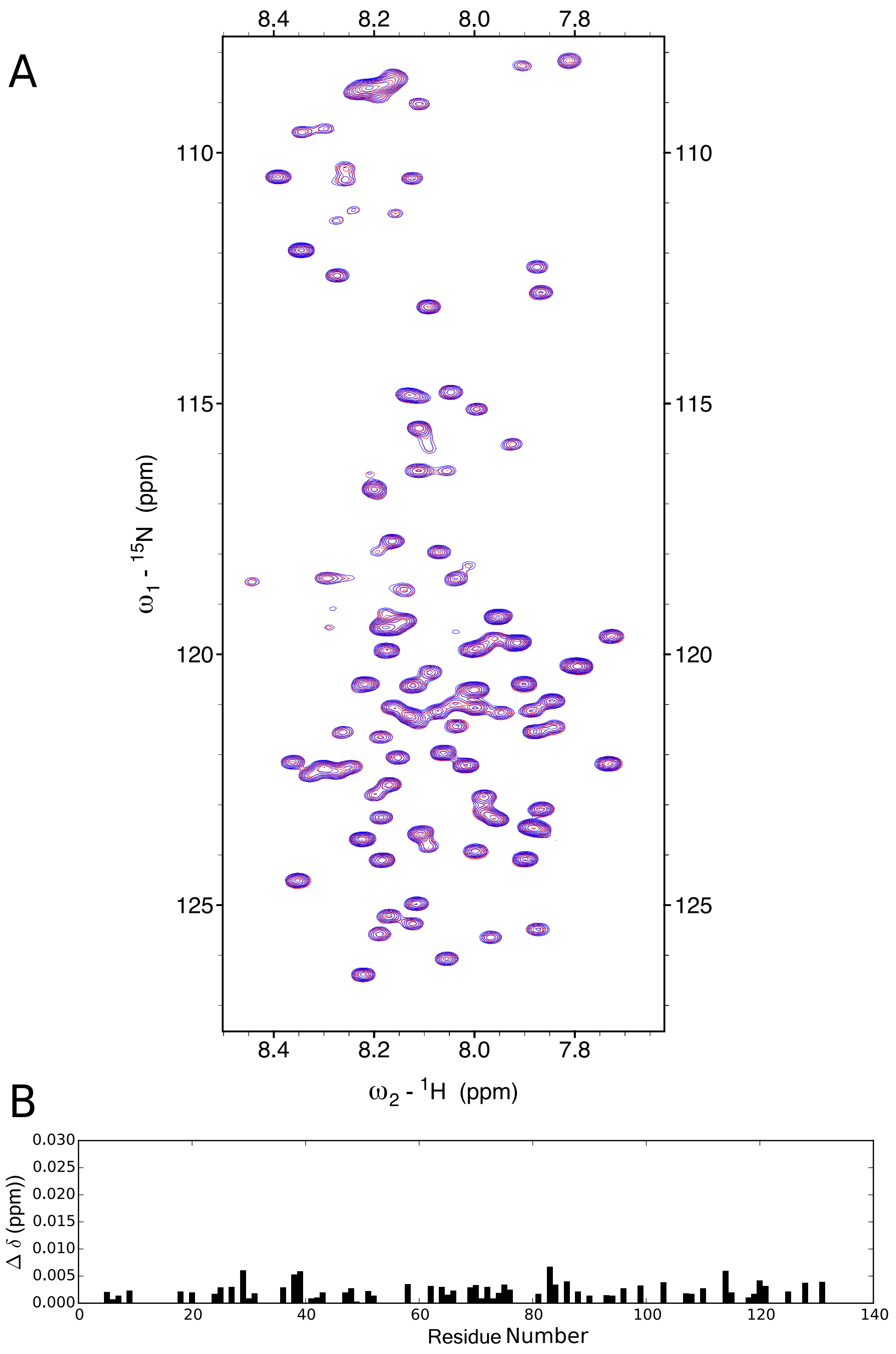

Supplementary Figure S1 – Excess CsgC causes no significant changes in CsgA chemical shifts. (A) ^{1}H-^{15}N HSQC NMR spectrum of 80 μM CsgA (Blue), and with unlabelled CsgC added at concentration of 500 μM (red). **(B)** Mapping of chemical shift perturbations caused by addition of CsgC.

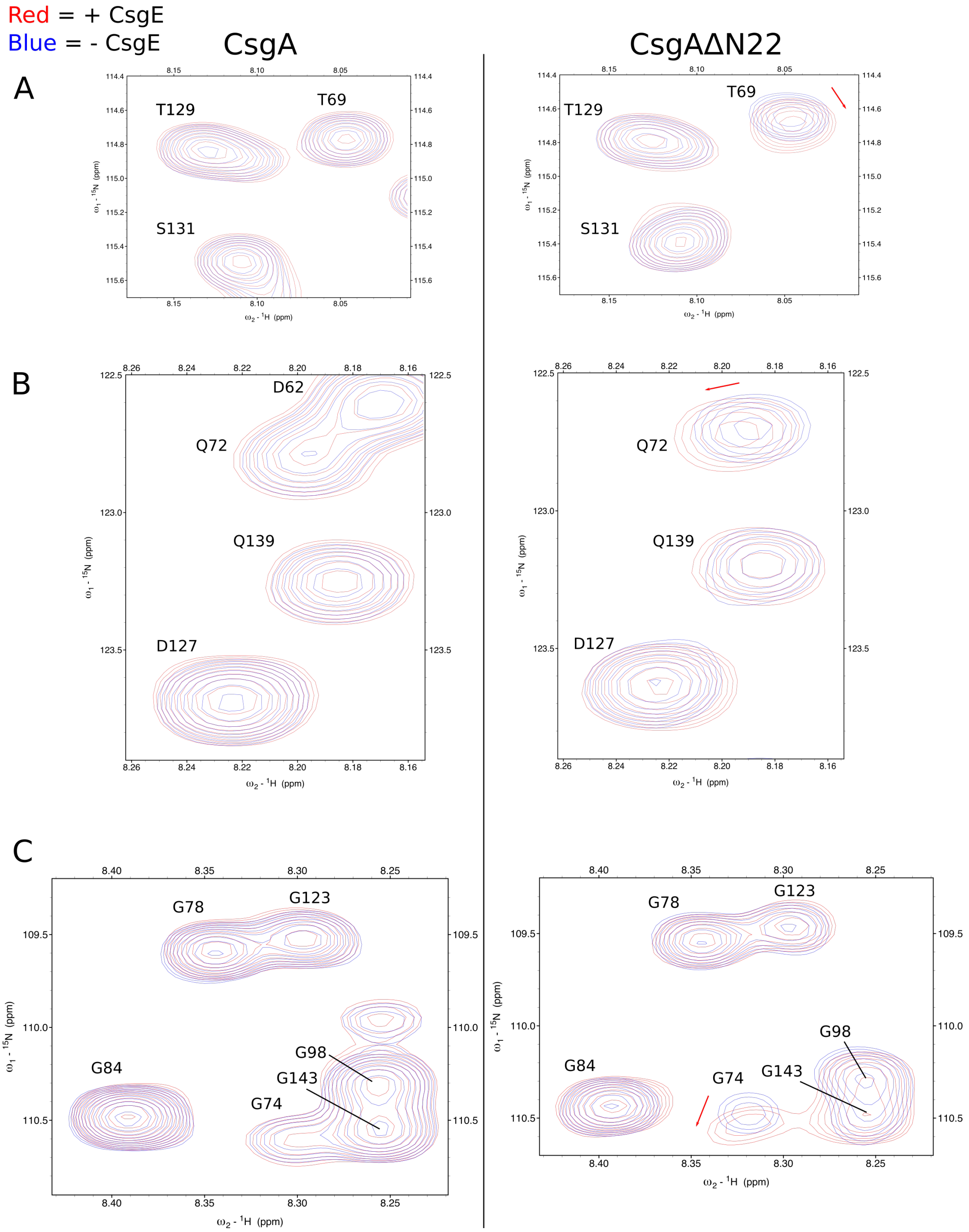

Supplementary Figure S2 – CsgE induces significant chemical shift perturbations only in $CsgA_{\Delta N22}$. Left panel displays overlay of ^{15}N-labelled CsgA and unlabelled W48A/F79A CsgE ^{15}N-HSQC spectra. Right panel displays overlay of ^{15}N-labelled $CsgA_{\Delta N22}$ and unlabelled W48A/F79A CsgE ^{15}N-HSQC spectra. Significant perturbations are observed in T69 **(A)**, Q72 **(B)**, G74 **(C)**. Red contours relate to spectra including CsgE, whilst blue are in absence of CsgE. Red arrows detail the direction of chemical shift perturbation.

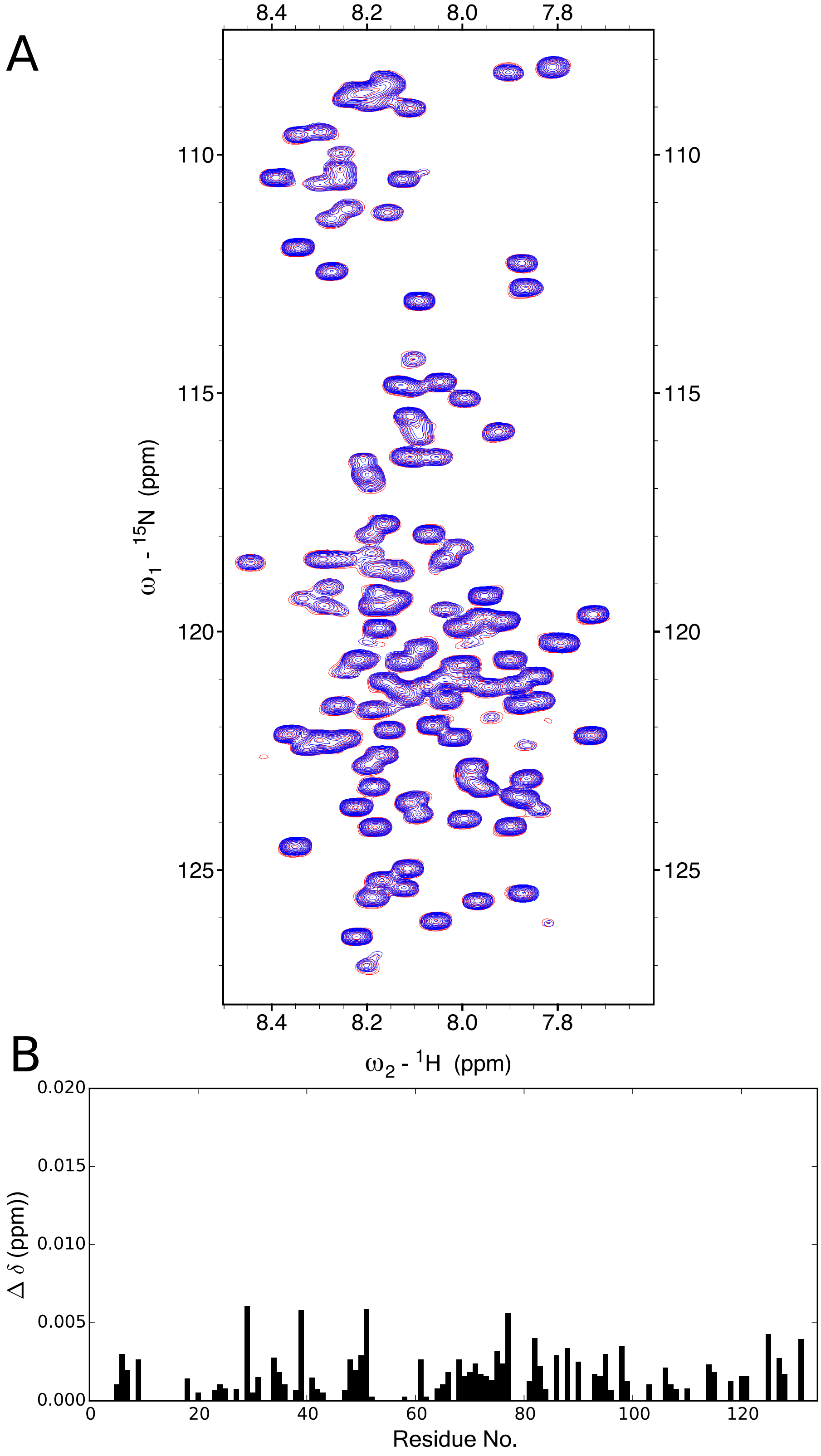

Supplementary Figure S3 – Presence of CsgE does not affect chemical shifts of full length CsgA
(A) ^{1}H-^{15}N HSQC NMR spectrum of 100 µM CsgA (Blue), and with unlabelled CsgE added at concentration of 400 µM. **(B)** Mapping of chemical shift perturbations caused by addition of CsgE.
